# Relevance of chemistry to white biotechnology

**DOI:** 10.1186/1752-153X-1-17

**Published:** 2007-06-20

**Authors:** Munishwar N Gupta, Smita Raghava

**Affiliations:** 1 Chemistry Department, Indian Institute of Technology Delhi, Hauz Khas, New Delhi, 110 016, India

## Abstract

White biotechnology is a fast emerging area that concerns itself with the use of biotechnological approaches in the production of bulk and fine chemicals, biofuels, and agricultural products. It is a truly multidisciplinary area and further progress depends critically on the role of chemists. This article outlines the emerging contours of white biotechnology and encourages chemists to take up some of the challenges that this area has thrown up.

## 

It is curious how new terms in science get coined: some terms appeal to the imagination of a large set of people and then a new area starts. Often, at the beginning of this process, people are not necessarily clear as to what the area includes or excludes. Increasingly, these 'new' areas attract people from different disciplines. A cross-fertilization of ideas and the pursuit of targets using tools from various existing areas hasten the development of the new area. The last few years have seen the increasing use of the word "white biotechnology" in the literature [1-4] (a search with "White biotechnology" on google gave 360,000 entries). It is not widely appreciated that chemists may have an important role to play if "white biotechnology" has to meet its challenges and deliver on its promises. An open access journal like *Chemistry Central Journal *(which aims to cover all of chemistry, including the interface between chemistry and life sciences) is perhaps an appropriate forum to talk about this fast emerging discipline in order to accelerate the involvement of chemists in this interesting area.

So, what is white biotechnology? Three terms related to biotechnology are in use [5]. Red biotechnology deals with the production of high value products (e.g., pharmaceutical proteins), green biotechnology covers the use of plants in biotechnology [6], whilst white biotechnology, involves the use of biotechnology in the production of bulk and fine chemicals [7] such as amino acids, vitamins, antibiotics, enzymes, drugs, organic acids and polymers [8,9]. It is appropriate to consider white biotechnology as green chemistry carried out using biotechnology tools [10].

Many people consider green chemistry and industrial biotechnology to be synonyms of white biotechnology. The laudable aim of white biotechnology is to create a sustainable society. While there has been occasional conflict between scientists and environmentalists, white biotechnology should lead to a synergy between these two groups of people. This is because white biotechnology focuses on the development of clean bioprocesses that should lead to "reductions in green house gas emissions, energy and water usage" [10]. An estimate by McKinsey & Company shows that biotechnology could be applied in the production of 10–20% of all chemicals sold by the year 2010. The study predicts that this will be motivated by both cost reduction as well as the promise of additional revenues (via new products and value added processes) [10]. Globally the chemical industry has about 10 million employees and a combined turnover of some €1300 billion [11]. In addition to being a huge source of pollutants, the industry depletes natural resources. The following are some of the key areas in which white biotechnology has shown considerable promise and wherein chemists and biochemists are expected to play an increasing role.

## Biomaterials

The demand for biodegradable polymers has grown at a rate of 20–30% per year [12]. The market segments include textiles, computers, mobile phones, gardening, packaging and flushable hygiene products. Poly(hydroxyalkanoates) and starch-based materials are the better known important examples of such materials. DuPont's Sorona™ is based on 1,3-propanediol, which in turn is produced from corn sugar. NatureWorks™ uses lactic acid that is again produced from the fermentation of corn sugar [13]. It is envisaged that future plastics would come from sugars, starch, cellulose and vegetable oils [12]. The underperformance of many bioplastics has delayed their wider adoption. The synergy between polymer chemists and biotechnologists should be able to meet this twin challenge of innovative production routes and product improvement.

Improved gene therapy strategies, drug delivery vehicles, biosensors, molecular gates and switches and control in microfluidics [14] are other areas that constitute an exciting interface between polymer chemists and white biotechnology.

## Biorefinery

While it all started with biofuels, namely, bioethanol and biodiesel, the concept of using renewable feedstock to form products that will replace petroleum-based products is catching on. Corns stover and energy crops like switch grass, miscanthus, energycane, and giant reed have shown considerable promise as renewable feedstock [15]. Conversion of starch/lignocellulosic material to sugars for subsequent fermentation to produce ethanol is receiving a lot of attention in terms of investment by both governments and industry. Efficient chemical pretreatment of feedstock before bioconversion is a challenge for chemists, which if met successfully would result in a quantum jump in our capabilities to produce ethanol from biomasses. The other biofuel, bodiesel, is obtained from the conversion of oils/fats to alkyl esters, which perform as well as diesel. Obtaining biodiesel from the inedible oils, such as from *Jatropha*, is being looked at by developing countries to meet their energy requirements [16]. There are some questions over whether switching over to biodiesel may really be a green option. However, the consideration of energy security is going to be an overriding factor.

Another new term, biorefinery, is attracting much attention. The US Department of Energy defined biorefinery as "an overall concept of a processing plant where biomass feedstock is converted and extracted into a spectrum of valuable products" [17]. To illustrate the scope, a study has identified 30 building blocks that can serve as intermediates in chemical industry. The list includes carboxylic acids, amino acids, furfural, sorbitol, and glycerol. In another study, production of wax esters in crambe is being carried out to obtain biolubricants for various applications such as engine oils, transmission oils, gear oils, hydraulic oils, and industrial greases [18].

## Obtaining and tailoring biocatalysts

Chemical industry uses 'fire and sword' chemistry to obtain desired chemicals [19]. In several cases the use of biocatalysts constitutes a viable green option. Enzymes can be used in the areas of food processing, textiles, detergents, edible oil extraction, leather processing, the restoration of old paintings, biofuel production and organic synthesis [20]. Recombinant DNA chemistry has paved the way for producing very large number of proteins/enzymes, while molecular biology has also made it possible to obtain enzymes from microbes that cannot even be cultured. This constitutes another recent approach called metagenomics [21]. These advances in upstream technology have resulted in efforts to develop efficient downstream strategies for these products, many of which exploit affinity-based strategies [22]. The search for economical and robust affinity ligands and the synthesis of biomimetic ligands has required that chemists use molecular modeling/docking techniques. Many approaches like site directed mutagenesis and directed evolution are being widely used to alter the stability and catalytic specificity of the enzymes [19]. It is often not appreciated that chemistry has played a pivotal role in the development of many of these technologies.

Much of the organic synthesis using enzymes is carried out in nearly anhydrous organic solvents or solvent free media [23]. Room temperature ionic liquids have more recently emerged as another nonaqueous medium, which, in view of their low vapour pressure, are viewed as 'green solvents' [24]. Chemical modification by bifunctional reagents can produce biocatalyst preparations such as CLEC™, CLEA™. These reusable, robust biocatalysts have been usefully employed for biotransformations in aqueous as well as nonaqueous media [25]. Microwave assisted synthesis is now considered an integral part of green chemistry, with some early work showing that microwave assisted enzymology may be an underexploited approach [26].

To sum up, a large part of white biotechnology represents a confluence of chemistry and biotechnology. It is not unlikely that chemists who use their expertise in this area may find it a rewarding experience. I do hope that those who do would choose *Chemistry Central Journal *to share this experience with others.
